# Mobile Cone-Beam CT-Assisted Bronchoscopy for Peripheral Lung Lesions

**DOI:** 10.3390/diagnostics13050827

**Published:** 2023-02-21

**Authors:** Moiz Salahuddin, Sami I. Bashour, Asad Khan, Gouthami Chintalapani, Gerhard Kleinszig, Roberto F. Casal

**Affiliations:** 1 Department of Pulmonary Medicine, The University of Texas MD Anderson Cancer Center, Houston, TX 77030, USA; 2 Siemens Medical Solutions USA Inc., Malvern, PA 19355, USA; 3 Siemens Healthcare GmbH, 91301 Forchheim, Germany

**Keywords:** cone beam CT, bronchoscopy, peripheral lung tumors

## Abstract

Peripheral bronchoscopy with the use of thin/ultrathin bronchoscopes and radial-probe endobronchial ultrasound (RP-EBUS) has been associated with a fair diagnostic yield. Mobile cone-beam CT (m-CBCT) could potentially improve the performance of these readily available technologies. We retrospectively reviewed the records of patients undergoing bronchoscopy for peripheral lung lesions with thin/ultrathin scope, RP-EBUS, and m-CBCT guidance. We studied the performance (diagnostic yield and sensitivity for malignancy) and safety (complications, radiation exposure) of this combined approach. A total of 51 patients were studied. The mean target size was 2.6 cm (SD, 1.3 cm) and the mean distance to the pleura was 1.5 cm (SD, 1.4 cm). The diagnostic yield was 78.4% (95 CI, 67.1–89.7%), and the sensitivity for malignancy was 77.4% (95 CI, 62.7–92.1%). The only complication was one pneumothorax. The median fluoroscopy time was 11.2 min (range, 2.9–42.1) and the median number of CT spins was 1 (range, 1–5). The mean Dose Area Product from the total exposure was 41.92 Gy·cm^2^ (SD, 11.35 Gy·cm^2^). Mobile CBCT guidance may increase the performance of thin/ultrathin bronchoscopy for peripheral lung lesions in a safe manner. Further prospective studies are needed to corroborate these findings.

## 1. Introduction

Lung cancer is the number one cause of cancer-related mortality for both men and women throughout the world [1]. Five-year survival in lung cancer is largely dependent on the stage of the disease at the time of diagnosis, ranging from 55% in early stages to 4% in metastatic disease [2]. Thus there is an effort to detect lung cancer at an early stage with annual screening by low-dose chest CT [3]. The combination of lung cancer screening with low-dose chest CT and the wide-spread use of chest CT scans has led to an increase in the number of detected lung nodules.

Diagnosis of peripherally located lung nodules can be achieved with a variety of techniques: bronchoscopy, CT-guided transthoracic needle biopsy, and video-assisted thoracoscopic surgery (VATS) [4]. With a favorable safety profile and the ability to provide nodal staging of lung cancer when indicated, the use of bronchoscopy to diagnose peripheral lung tumors has substantially grown in the past two decades. Navigational technologies (electromagnetic and non-electromagnetic) and, more recently, robotic bronchoscopy, have been added to the bronchoscopists’ armamentarium. These technologies have been demonstrated to provide a greater diagnostic yield than the use of thin/ultrathin (T/UT) bronchoscopy with radial-probe endobronchial ultrasound (RP-EBUS) [5,6,7,8,9]. However, these technologies are costly (requiring capital equipment purchase and disposables for every case) and not widely available outside the United States of America.

As we have previously described, there are three main phases during peripheral bronchoscopy: navigation, confirmation, and acquisition [10]. For long, RP-EBUS has been the sole method to confirm that a target has been reached during bronchoscopy. Unfortunately, RP-EBUS is not an accurate method of target confirmation, as has been highlighted by recent work from our group demonstrating that atelectasis occurs very commonly during bronchoscopy under general anesthesia, and can easily create false-positive RP-EBUS images [11,12]. Thus the interest in the use of cone-beam CT (CBCT) guidance, in particular for the phase of confirmation, to compensate for the suboptimal performance of RP-EBUS [11,12,13,14,15,16,17]. Multiple studies have demonstrated that CBCT can increase the yield of different navigational techniques [11,14,15,16,17]. However, most of these studies were performed with “fixed” CBCTs. This type of CBCTs is typically located outside of the bronchoscopy suites (interventional radiology suites, hybrid operating rooms), and most pulmonologists do not have access to it. Hence, the interest in “mobile” CBCT (m-CBCT)—a more affordable alternative which has the advantage of being able to be shared amongst multiple hospital service lines, can be easily “rolled” in and out of most bronchoscopy suites and can provide both two dimensional (2D) and three dimensional (3D) images [13]. The combination of m-CBCT along with the readily available T/UT bronchoscopes and RP-EBUS has been briefly described in case reports and a small case series [18,19]. Data on diagnostic performance and radiation exposure are needed before adopting this new imaging technology to guide our bronchoscopies. Here, we report our initial experience with the use of m-CBCT in combination with T/UT bronchoscopy and RP-EBUS for patients with peripheral lung lesions, and its efficacy and safety.

## 2. Materials and Methods

### 2.1. Study Center and Population

After obtaining IRB approval (2021-0268), consecutive patients were captured retrospectively through electronic medical records at The University of Texas MD Anderson Cancer Center between June 2020 and March 2021. Bronchoscopies performed for the diagnosis of peripheral lung lesions (located in the outer two-thirds of the lungs) with the combination of thin (BF-P190F; Olympus, Tokyo, Japan) or ultra-thin scopes (BF-MP190F; Olympus, Tokyo, Japan), RP-EBUS (UM-S20-17S, Olympus, Tokyo, Japan), and m-CBCT (Cios Spin, Siemens Healthineers, Forchheim, Germany) were included in this analysis.

### 2.2. Procedures

**Figure 1:**
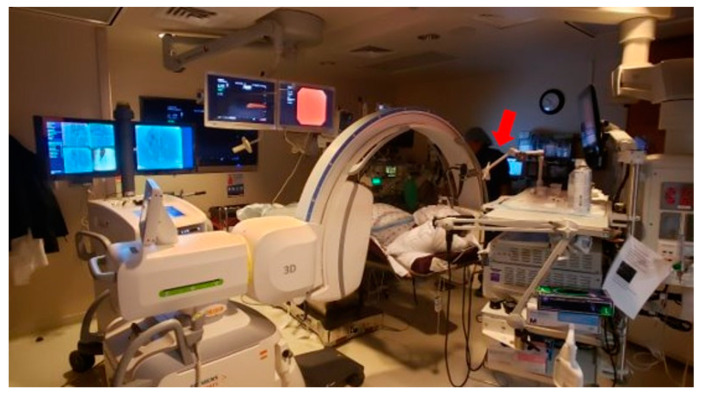
Bronchoscopy suite set up with m-CBCT. The red arrow corresponds to the metallic arm holding the bronchoscope during cone beam CT scan.

Bronchoscopies were performed under general anesthesia, without a ventilatory strategy to prevent atelectasis (data not available at that time), through a laryngeal-mask airway (LMA) in most cases, with 100% FiO_2_ and 0–5 cmH_2_O of positive-end of expiration pressure (PEEP). Five different physicians performed bronchoscopies. These physicians operated the m-CBCT C-arm themselves. A combination of sampling tools including needle (21 G PeriView FLEX needle; Olympus, Tokyo, Japan), cytology brush (BC-202D, Olympus, Tokyo, Japan), and biopsy forceps (FB-231D, Olympus, Tokyo, Japan) was utilized at the discretion of the operators. Broncho-alveolar lavage was performed when infection was suspected. Rapid on-site cytology examination (ROSE) was present for all cases. The m-CBCT C-arm was utilized for both fluoroscopy imaging (2D) and CT scans (3D) at the discretion of the operator. Using its laser projection, the m-CBCT C-arm was isocentered on the target lesion. The m-CBCT scan consisted of a 30 s spin, with the target nodule positioned in the center and C-arm rotating around the patient acquiring 400 projection images in fixed angular intervals that are reconstructed into multiplanar cross-sectional CT-like images. Each m-CBCT spin was performed with a ventilatory breath hold of 30 s (time required for spin). During spins, the bronchoscope was fixed in position with an articulating arm that attaches to the bronchoscopy boom (The Arm, Neuwave Medical Inc., Madison, WI, USA), so that all personnel could exit the room (except for one anesthesia staff that remained behind a protective shield) (Figure 1). Patients were recovered per standard of care and all patients underwent post-procedural chest X-ray to rule out pneumothorax at the end of the recovery period.

### 2.3. Study Definitions

Our primary endpoint was “diagnostic yield”, defined as the number of patients in whom diagnostic samples are obtained divided by the total number of patients undergoing bronchoscopy. Samples were considered diagnostic if they demonstrated malignancy or a benign (but abnormal) process. Samples showing blood, bronchial cells, macrophages, and non-specific acute or chronic inflammation were considered “non-diagnostic”. Benign tumors, specific infections or granulomatous inflammation were considered diagnostic (provided that further biopsies, surgery or clinical-radiographical follow-up agreed with this). All patients with samples that did not show malignancy were further assessed by either CT-guided fine needle aspiration (FNA), surgery, or radiographic follow-up at the discretion of their managing physicians. Chart records and radiographic follow-up were reviewed 18 months post-bronchoscopy.

The main secondary endpoints included sensitivity for malignancy, complications, and radiation exposure. The gold standard to calculate sensitivity for malignancy was either surgical pathology (from lung resection when available), CT-guided FNA, or 18-month clinical and radiographic follow-up. We defined sensitivity as true positives (TP)/true positives (TP) + false negatives (FN) with the disease being malignancy of any type. All bronchoscopic samples showing malignancy were considered TP. Cases where bronchoscopic samples were not diagnostic of malignancy and malignancy was later confirmed by either CT-guided FNA, surgery, or radiographical progression of disease were considered FN. Intra-bronchoscopy and post-bronchoscopy complications were extracted from medical records. Fluoroscopy time, number of m-CBCT spins, and radiation exposure associated with each were recorded. Radiation exposure measured as the dose area product (DAP) was defined as product of dose and beam area (Gy cm^2^) and it was measured using an ionization chamber placed between the X-ray tube/collimator setup and the patient. Other relevant data collected included demographics, patient characteristics, target characteristics (anatomic location, distance to the pleura, size, radiographic characteristics, presence of bronchus sign), and procedure characteristics (e.g., duration which was defined as first scope “in” to last scope “out” and concomitant mediastinal staging).

Descriptive statistics (frequencies, proportions, means, standard deviations, medians, and range) were provided for patient, target, procedure characteristics, and radiation exposure. Diagnostic yield and sensitivity for malignancy with 95% confidence intervals were reported. SAS 9.4 (SAS Institute INC, Cary, NC, USA) was used for data analysis.

## 3. Results

A total of 51 patients were included in the analysis. The mean target size was 2.6 cm (SD, 1.3 cm) and the mean distance to the pleura was 1.5 cm (SD, 1.4 cm) (see patient and target characteristics in Table 1). Procedure characteristics are depicted in Table 2. The most utilized sampling tool was the needle (94% of the procedures). The median procedural time was 85 min (range of 24–144 min), which included the time required for mediastinal staging which was performed in 33 patients (65%). The diagnostic yield was 78.4% (95 CI, 67.1–89.7%), and the sensitivity for malignancy was 77.4% (95 CI, 62.7–92.1%). Specific diagnoses are described in Table 3. Malignancy was detected in 24 patients and missed in 11 patients. Sixteen patients had benign diagnoses. The only recorded complication was one case of pneumothorax requiring a chest tube. The median fluoroscopy time was 11.2 min (range, 2.9–42.1) and the median number of CT spins was 1 (range, 1–5). The mean fluoroscopy time was 13.59 min (range, 2.9 to 42.1). Fluoroscopy and CBCT (3D spins)-associated radiation exposure is described in Table 4.

## 4. Discussion

This is the largest report on the specific combination of T/UT bronchoscopy, RP-EBUS, and m-CBCT, and the largest one to report radiation exposure associated with m-CBCT. When comparing with prior literature of UT/T bronchoscopy and RP-EBUS, the addition of m-CBCT seems to positively impact its results in terms of diagnostic yield and sensitivity for malignancy [6,7]. Procedures were safe (only one pneumothorax) with acceptable levels of radiation exposure (approximately two-thirds of DAP was secondary to fluoroscopy and one-third due to the m-CBCT spins).

Navigational bronchoscopy platforms, robotic bronchoscopy platforms, use of tomosynthesis, augmented fluoroscopy, and fixed CBCT are rapidly evolving and increasing our reach for peripheral lung lesions. Unfortunately, these pieces of equipment and the required disposables are too costly and not within reach to the majority of bronchoscopists world-wide. Because of this, it is imperative to improve the performance of the simpler and readily available tools such as the use of T/UT bronchoscopes. A large multicenter randomized controlled trial of standard fluoroscopy guided bronchoscopy versus thin bronchoscope with RP-EBUS by Tanner and coworkers reported a fair diagnostic yield of 49% with thin bronchoscopy/RP-EBUS [6]. This same study showed one of the greatest gaps reported in the literature between navigation and diagnostic yield. Of 179 patients who underwent RP-EBUS either because of randomization or subsequent crossover, 174 (97%) had ultrasound “confirmation” of lesion localization, with a concentric image seen in 113 (65%). Yet, diagnostic yield was 50% for concentric and 31% for eccentric lesions. A potential partial explanation for this enormous gap between navigational yield and diagnostic yield is the “pseudo-confirmation” of target reach given by falsely positive RP-EBUS images (attributed to atelectasis, clotting, etc.). Of course, factors associated with the sampling tools they utilized may have had a role as well in this enormous gap. As mentioned before, electromagnetic navigational bronchoscopy has also demonstrated a diagnostic yield around 70%, close to what we have shown in this study [8]. Nevertheless, from the cost-effective aspect, the need of specific disposable navigational tools, and sometimes the need for repeat chest CT with specific slice thickness, makes these navigational techniques less feasible for many bronchoscopists throughout the world. Lastly, transthoracic needle aspiration—with a higher yield, lower cost, and higher rate of pneumothorax—is unable to provide nodal staging, making it a less attractive option for patients with suspected or known lung cancer.

The need for a more precise and certain method for confirmation of navigation success has led our group and many others to investigate the additional value of CBCT guidance during peripheral bronchoscopy. One of the initial studies to combine T/UT bronchoscopes with fixed CBCT came from our own group [11]. We performed a prospective pilot study (*n* = 20 patients) in which we assessed the effect on navigational and diagnostic yield provided by the addition of fixed-CBCT imaging to the standard 2D fluoroscopy and T/UT bronchoscopy combination. CBCT imaging resulted in a 25% absolute increase in navigational yield (from 50 to 75%, *p* = 0.02), and 20% absolute increase in diagnostic yield (from 50 to 70%, *p* = 0.04). The median fluoroscopy time was 8.6 min (range, 5–15.4), and the median number of CT scans was 1.5 (range, 1–2). The mean DAP from the total exposure was 64.57 Gy·cm^2^ (range, 6.14–114.89 Gy·cm^2^), and originating in the CBCTs 50.45 Gy·cm^2^ (range, 5.43–66.75 Gy·cm^2^). Both diagnostic and navigational yield and overall radiation exposure figures were not that distant from our current study. Of note, opposite to our current study, most radiation exposure originated from the CBCTs, instead of originating from 2D fluoroscopy. A more recent report of T/UT bronchoscopy along with fixed CBCT and “augmented fluoroscopy” (Artis zeego, Siemens Healthineers, Forchheim, Germany) is that of DiBardino and coworkers [17]. This study retrospectively analyzed three cohorts of patients: UT bronchoscope + fixed CBCT + RP-EBUS (*n* = 30); thin or therapeutic bronchoscope + fixed CBCT + RP-EBUS (*n* = 27); thin or therapeutic bronchoscope + RP-EBUS (*n* = 59). Diagnostic yields were 85.0% (95% CI, 68.6% to 100%), 68.3% (95% CI, 50.1% to 86.6%), and 44.5% (95% CI, 31.0% to 58.0%), respectively. The median pulmonary lesion diameter was 1.95 cm (interquartile range, 1.5 to 2.75 cm). Virtual navigational bronchoscopy (VNB) (Archimedes, Broncus Medical Inc., San Jose, CA, USA) was most utilized in the thin or therapeutic bronchoscope + RP-EBUS group (45.8% vs. 18.5%, *p* = 0.02; 45.8% vs. 13.3%, *p* = 0.002) compared with the thin or therapeutic bronchoscope + fixed CBCT + RP-EBUS and UT bronchoscope + fixed CBCT + UTB + RP-EBUS groups, respectively. They reported a median radiation dose in the fixed-CBCT groups of 70.42 Gy·cm^2^ (IQR: 42.49 to 99.70). Their study differed from our current one in multiple ways: they utilized augmented fluoroscopy with their fixed CBCT in all cases and they allowed for virtual navigational bronchoscopy. While their reported diagnostic yield is only slightly higher than ours, their mean radiation exposure is substantially higher. This could be explained, in part, by the fact that the operators obtained an extra scan to corroborate tool in lesion.

Data on m-CBCT guiding T/UT bronchoscopy are truly scant. The first report is in the form of a “research letter” by Avasarala and coworkers [19]. They reported on the use of an m-CBCT (Cios Spin, Siemens Medical Solutions, Malvern, PA, USA) as a guide for peripheral bronchoscopy in eight patients with an average lesion size of 2.6 cm (electromagnetic navigational bronchoscopy, superDimension Navigation System version 7.0; Medtronic, Minneapolis, MN, USA was used in two cases). Though results are restricted by the sample size, they reported a mean radiation exposure per procedure of 40.92 Gy·cm^2^, quite similar to ours. The only additional report on the m-CBCT guiding T/UT bronchoscopy combination (same m-CBCT system) that we could find in the literature is that of Sadoughi and coworkers, which consists of a case series of four patients [18]. The use of a different m-CBCT system, the O-arm O_2_ Imaging System (Medtronic, Minneapolis, MN, USA), was reported in combination with electromagnetic navigational bronchoscopy (EMN) (superDimension Navigation System version 7.0; Medtronic, Minneapolis, MN, USA), and RP-EBUS by Cho and coworkers [20]. In this very small report, the average nodule size was 2.1 cm, and only two of the six cases were diagnostic. Unfortunately, DAP was not reported in this case series for comparison, they only reported an estimated average effective dose per 3D spin of 3.73 mSv.

Mobile-CBCT guidance use has been reported with more sophisticated bronchoscopy technologies such as electromagnetic navigation and robotic bronchoscopy [21,22]. In a very interesting brief research report, Chan and coworkers reported the use of EMN with both mobile-CBCT (Cios Spin, Siemens Healthineers, Forchheim, Germany) and floor-mounted CBCT (syngo DynaCT^®^ of Artis Zeego by Siemens Healthineers) in a hybrid operating room [21]. They utilized this combination for a total of 11 procedures: 5 diagnostic biopsies, 4 transbronchial microwave ablations, and 2 cases of dye marking followed by surgery. They intentionally utilized both CBCT systems in order to compare image quality and ease of use. Navigation success was achieved in four out of five cases. The authors reported comparable ease-of-use, and they stated that the m-CBCT unit was able to identify most lung lesions, especially the larger and denser nodules. In two cases of ground glass opacities (8 and 13 mm in diameter), the authors describe that these lesions were faintly seen with m-CBCT and clearly seen with their floor-mounted counterpart. An elegant study by Reisenauer and coworkers reported the combination of m-CBCT (Cios Spin, Siemens Healthineers, Forchheim, Germany) and Shape-Sensing Robotic-Assisted Bronchoscopy (SSRAB) (Ion Endoluminal Robotic Bronchoscopy System, Intuitive Surgical, Sunnyvale, CA, USA) [22]. The objectives of this small prospective pilot study were to assess the ability of m-CBCT to demonstrate tool in lesion, to calculate CT-to-body divergence, and report diagnostic yield and radiation exposure. A total of 30 nodules were sampled, with a median size of 17.5 mm (SD, 6.8) in the largest dimension. The mean airway generation was 7, with a mean distance to pleura of 14.9 mm (range, 1–45.8 mm). Bronchus sign was present in 40% of patients. In 100% of procedures, the proceduralist was able to navigate to the lesion, with a mean number of m-CBCT spins of 2.5 (SD of 1.6). Nineteen (63.3%) cases had an eccentric RP-EBUS signal, 13.3% had a concentric signal, and 23.3% had no signal with RP-EBUS. The total mean fluoroscopy time was 8.7 min (range, 2–27 min) and the total mean DAP was 50.3 Gycm2 with an average of 2.5 spins overall for all cases. The authors reported a diagnostic yield of 93.3%, with a true positive rate 73.3% (22/30 cases) with a 6.7% false negative rate (2/30 cases), and an overall sensitivity for malignancy of 91.7%. These promising results once again show how image guidance with m-CBCT can improve the navigational and diagnostic yield of the most sophisticated bronchoscopy technologies, such as this new robotic platform. The fact that a mean of 2.5 spins were necessary to reach lesions and obtain diagnosis shows that in many of these cases success would have not occurred without this image guidance helping correct the CT to body divergence.

With our aging population and the wide-spread use of chest CT, we are bound to diagnose more and more often early-stage lung cancer in medically inoperable patients. In the past few years, bronchoscopists have ventured into the field of therapeutic bronchoscopy with the development of several different modalities for bronchoscopic ablation of lung cancer or lung metastases [23]. While these different ablative techniques are still at a very early investigational level, they do have something in common, which is the need for a real-time imaging modality that can show the location of the ablative probe with respect to the target, vital structures, and the pleura [10]. Whereas fixed-CBCT has been utilized in most reported experiments, Chan and coworkers described the use of m-CBCT in four cases of transbronchial microwave ablations, and Chen and coworkers described another case of transbronchial microwave ablation of a 15 mm left upper lobe ground glass opacity [21,24]. With comparable image quality, m-CBCT will likely allow more centers throughout the world to slowly adopt bronchoscopic ablative techniques once they are demonstrated to be safe and effective.

Our study has a few limitations. At the time these bronchoscopies were done, not all m-CBCT images were being recorded and some proceduralists were not obtaining an extra spin to document tool in lesion (TIL). Thus, we do not have information with regards to TIL, which we believe is key when utilizing CBCT guidance. As described in the methods, ROSE was utilized in all cases. ROSE may not be available in some institutions, and it may have increased our diagnostic yield. Our clinical and radiographic follow-up for suspected benign lesions was 18 months. Though we considered this appropriate and in line with other publications, slow or non-growing adenocarcinomas of the lung could be missed with this observation period.

## 5. Conclusions

Bronchoscopic navigational platforms have been shown to improve the yield of peripheral bronchoscopy but they are also costly and not available world-wide. In lieu of the new evidence suggesting that RP-EBUS may be an inaccurate confirmatory tool for peripheral bronchoscopy, image guidance with m-CBCT may be key to improving the performance of the widely available thin and ultrathin bronchoscopes. Our report, the largest of its kind, suggests that m-CBCT guidance may increase the performance of thin and ultrathin bronchoscopes for peripheral lung lesions in a safe manner. Further prospective studies are needed to corroborate these findings.

## Figures and Tables

**Table 1:** Patient and target characteristics.

Characteristics	*N* = 51
**Gender** - Female - Male	26 (51) 25 (49)
**Smoking History** - Never - Ex-smoker - Current	18 (35) 24 (47) 9 (18)
**Prior Malignancy** - Lung cancer - Others	10 (20) 20 (40)
**ECOG** Median (range)	1 (0–2)
**ASA Score** Median (range)	3 (2–4)
**Target Size (cm)** Mean (SD)	2.6 (1.3)
**Target Characteristics** - Solid - Semi-solid	37 (73) 14 (27)
**Target Location** - Right Upper Lobe - Right Middle Lobe - Right Lower Lobe - Left Upper Lobe - Left Lower Lobe	21 (41) 3 (6) 5 (10) 17 (33) 5 (10)
**Bronchus Sign**	39 (75)
**Distance to Pleura (cm)** Mean (SD)	1.5 (1.4)

(ECOG = Eastern Cooperative Oncology Group; ASA = American Society of Anesthesiology; SD = standard deviation).

**Table 2:** Procedure Characteristics.

Procedure Characteristics	*N* = 51
**Artificial Airway** - Laryngeal Mask Airway - Endotracheal Tube	44 (86) 7 (14)
Sampling Tools * - Needle - Forceps - Cytology Brush	48 (94) 29 (57) 20 (21)
**Radial-Probe EBUS** - Eccentric view - Concentric view - Image not recorded	29 (57) 13 (25) 9 (18)
**Presence of Atelectasis by CT**	24 (47)
**Procedure Time (min)** Median (range)	85 (24–144)
**Mediastinal Staging**	33 (65)
**Fluoroscopy Time (min)** Median (range)	11.2 (2.9–42.1)
**Number of CBCT Spins** Median (range)	1 (1–5)

* More than one tool was utilized in most cases. RP-EBUS = radial-probe endobronchial ultrasound; CBCT = Cone-Beam Computed Tomography.

**Table 3:** Diagnosis Obtained with Bronchoscopy.

Malignancy (*n* = 24)		Benign (*n* = 16)		Non-Diagnostic Sample (*n* = 11)	
**Lung Cancer** Adenocarcinoma Squamous Cell Carcinoma Non-Small Cell (not specified) Small Cell Lung Cancer Carcinoid **Others** Lymphoma Melanoma Renal Cell Carcinoma	14 3 1 1 1 2 1 1	**Infectious/Presumed Infectious** Granulomas ᵻ Aspergilloma Coccidioidomycosis Necrotizing pneumonia Organizing pneumonia ᵻ Histoplasmosis Staphylococcus Aureus pneumonia **Others** Hamartoma Post-Radiation Scar ᵻ	4 2 2 2 2 1 1 1 1	**Follow-Up Diagnosis** Lung cancer Prostate cancer Lymphoma Granulomatous inflammation Subsequently resolved opacity	5 1 1 1 3

ᵻ Confirmed with CT-FNA, resolved or improved during 18-month follow-up.

**Table 4:** Fluoroscopy and Cone-Beam CT-Associated Radiation Exposure.

	Total Fluoroscopy Time (min)	Number of 3D Spins	Dose Area Product from Total Exposure (Gy·cm^2^)	Dose Area Product from 3D Spins (Gy·cm^2^)
**Mean**	13.59	1.82	41.92	11.35
**Std**	7.91	1.01	26.19	6.24
**Min**	2.9	1	9.10	4.38
**Max**	42.1	5	113.08	28.53

## Data Availability

Not applicable.
